# How much centralization of critical care services in the era of telemedicine?

**DOI:** 10.1186/s13054-019-2705-1

**Published:** 2019-12-26

**Authors:** Marlies Ostermann, Jean-Louis Vincent

**Affiliations:** 1 Department of Critical Care, King’s College London, Guy’s and St Thomas’ NHS Foundation Trust, London, SE1 7EH UK; 2 Department of Intensive Care, Erasme University Hospital, Université Libre de Bruxelles, Route de Lennik 808, 1070 Brussels, Belgium

**Editorial**

The goal of modern health care is to improve outcomes and reduce costs. Centralisation, defined as the reorganisation of healthcare services into fewer specialised units, is one of the common strategies. The rationale is that increasing the volume and variety of cases promotes the development of highly specialised services, increases experience and efficiency, facilitates training, limits costs and reduces clinical variability [1–3]. The notion of focussing on volume to promote specialist expertise is well established in surgery. There is a clear association between volume of surgical cases and survival, even if workload increases [1]. Obvious examples are large cardiovascular units and trauma centres. The reasons for better outcomes are multifactorial, including expert teams, a high-level infrastructure with evidence-based protocols and standardised governance processes, state-of-the-art diagnostic tests and therapies, and cost-effective purchasing (Table 1).

**Table 1:**
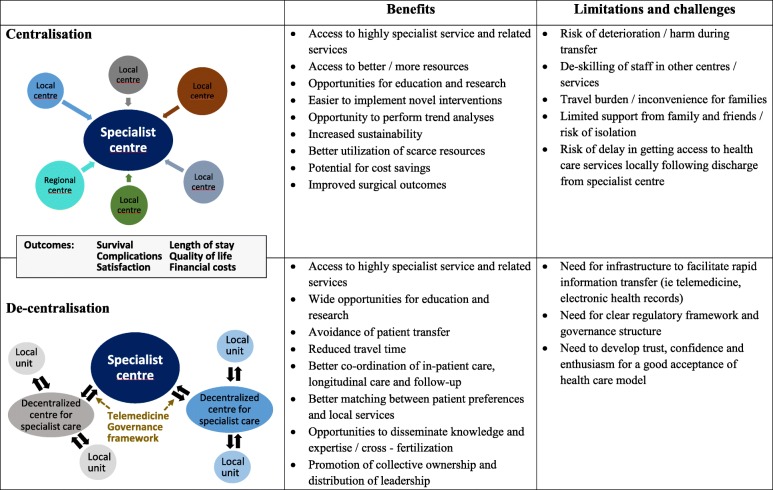
Benefits and challenges of centralised and de-centralised provision of critical care

Critical care medicine is a complex, expensive and resource intensive specialty where centralisation has also received attention. A retrospective study of >20,000 mechanically ventilated, non-surgical adult patients concluded that ICU and hospital mortality were significantly lower in high-volume hospitals [4]. The “Conventional ventilatory support versus Extracorporeal membrane oxygenation (ECMO) for Severe Adult Respiratory failure” (CESAR) trial showed that outcomes were better in all patients transferred to the specialist unit regardless of whether they received ECMO or not [5]. Neurocritical care units have been shown to improve patient outcomes and reduce mortality, resource utilisation and costs compared to district hospitals [6]. Apart from specialist-led care, rapid access to neurosurgical intervention plays a role. Finally, a review of centralised paediatric critical care in Australia revealed that the odds ratio of mortality in the UK versus Australia was 2.09 [7]. The authors estimated that 453 deaths a year in the UK could be avoided if all children requiring mechanical ventilation for >12–24 h were transferred to specialist paediatric ICUs.

However, the association between volume and outcomes is not consistently seen. Data from 2812 US hospitals showed that quality of care for elderly patients with pneumonia was lower among hospitals with the highest rates of ICU admission [8]. Similarly, an analysis of > 18,000 ECMO patients revealed that mortality was higher in high-volume compared to low-volume centres [9]. Whether this represents selection bias, differences in criteria for applying ECMO or any other variation in practice is unclear.

Centralisation of limited resources has other unpredictable negative effects which can be broadly categorised into factors related to the geographical distance between centres, transport, the effects on staff in non-specialist centres, and the psychological impact on the patient and their relatives. Serious in-transit critical events may occur, including equipment failure and technical problems [10–12]. A review of 5144 urgent land transports revealed that critical events occurred in approximately 1 in 15 transports [12]. Hypotension was the most common incident. An observational study of > 10,000 patients with potentially life-threatening conditions showed an association between journey distance to hospital and mortality after adjustment for age, sex, clinical category and illness severity [10]. A 10-km increase in distance was associated with a 1% absolute increase in mortality. In contrast, a Canadian retrospective case-cohort study did not find an association between duration of transport and hospital mortality [13]. Instead, a longer time spent by paramedics at the sending hospital was associated with shorter length of stay in the referring hospital.

At an institutional level, centralisation may lead to a reduction in available specialists in regional centres and the closure of specialty programmes, resulting in reduced job satisfaction and staff morale [11]. Another drawback is the impact on families and relatives, together with longer travel times and increased costs. Furthermore, patients are removed from their local networks which makes it more challenging to organise long-term support and chronic disease management after critical illness (i.e. social service, psychological follow-up).

Little is known about the patient’s perspective. Work by the Swedish National Board of Health and Welfare concluded that patients valued quality of care and treatment outcomes as the most important factors regarding centralisation of healthcare; continuity of treatment and a well-functioning care pathway were also very important [14]. To date, the discussions related to the benefits of centralisation versus de-centralisation have focused mainly on the impact on mortality and healthcare costs but less on other outcomes like ICU-acquired infections, patient and family satisfaction, risk of post-traumatic stress and quality of life.

There are good reasons to explore new ways of delivering high quality patient-centred affordable care. Decentralisation of health systems is a potential alternative strategy (Table 1). It is considered to improve efficiency and quality of service as well as promoting accountability, local governance and sharing of knowledge and expertise. This shift towards integration of specialist expertise in the local environment holds great promise to improve the patient experience and facilitate training while keeping healthcare costs under control. Through telemedicine and robust electronic medical record platforms, distant care providers can interact with the clinical team and also potentially with the patient and their family so that direct round-the-clock access to specialist expertise is provided. Such a system can promote training, dissemination of knowledge and cross-fertilisation but whether it reduces variability in clinical care and improves patient outcomes and staff morale is unclear. Among the key concerns about telemedicine are the need to maintain privacy, confidentiality and security of personal data, and the risk of incorrect diagnosis or treatment. In 2019, the General Medicine Council UK commissioned Europe Economics to review the regulatory approaches to telemedicine [15]. The panel concluded that telemedicine needed to (i) deliver the same standard of care as that of face-to-face healthcare; (ii) ensure confidentiality, safety and security of the exchanged information; (iii) uphold patient safety where prescribing may be contemplated, and (iv) include the patient’s consent. The panel highlighted that the requirement to obtain patients’ consent was covered by only 11 jurisdictions across the world.

In conclusion, critical care medicine of the future is likely to look very differently and determining the extent of centralisation versus de-centralisation will be necessary. As the utilisation of new technologies expands, the regulatory framework needs to evolve, too.

## Data Availability

Not applicable.
